# Prevalence of Self-Reported Temporomandibular Disorder Symptoms in Patients With Obstructive Sleep Apnea Based on the Fonseca Anamnestic Index: A Cross-Sectional Multicentric Study

**DOI:** 10.7759/cureus.105565

**Published:** 2026-03-20

**Authors:** Abhinav Shekhar, Supriya Singh, Shitij Srivastava, Rashika Singh, Bhaskar Agarwal, Ajay Verma

**Affiliations:** 1 Department of Prosthodontics, Sardar Patel Post Graduate Institute of Dental and Medical Sciences, Lucknow, IND; 2 Department of Prosthodontics, King George's Medical University, Lucknow, IND; 3 Department of Respiratory Medicine, King George's Medical University, Lucknow, IND

**Keywords:** fonseca anamnestic index, obstructive sleep apnea, prosthodontics, respiratory medicine, temporomandibular disorder

## Abstract

Background

Obstructive sleep apnea (OSA) is a common sleep-related breathing disorder characterized by recurrent upper airway obstruction and fragmented sleep. Temporomandibular disorder (TMD) comprises musculoskeletal conditions affecting the temporomandibular joint and masticatory muscles, often presenting with pain and functional limitations. Both conditions share clinical features such as sleep disturbance and musculoskeletal discomfort, suggesting a possible relationship. However, the prevalence of TMD among patients with OSA and its association with clinical characteristics remains insufficiently explored.

Objectives

The main objective of this study is to determine the prevalence of symptoms suggestive of TMD among patients with severe OSA using the Fonseca Anamnestic Index (FAI) and to evaluate the association between TMD severity and selected demographic and clinical variables, including age, gender, body mass index (BMI), and OSA-related symptoms.

Methods

This cross-sectional, multicentric, observational study was conducted from August 2024 to August 2025 at the Department of Prosthodontics and Crown & Bridge, Sardar Patel Postgraduate Institute of Dental and Medical Sciences, Lucknow, India, in collaboration with the Departments of Prosthodontics and Crown & Bridge and Respiratory Medicine at King George’s Medical University, Lucknow, India. A total of 110 adults aged 18-60 years with polysomnography-confirmed severe OSA (apnea-hypopnea index ≥ 30 events/hour) were enrolled. Patients with prior OSA or TMD treatment, temporomandibular joint surgery or fracture, malocclusion, orthodontic history, recent analgesic use, neurological disorders, or unwillingness to provide consent were excluded. Symptoms suggestive of TMD were assessed using the FAI. Demographic, clinical, and OSA-related variables were recorded. Descriptive statistics and association analyses were performed to evaluate relationships between TMD severity and clinical parameters.

Results

The study population had a mean age of 48.28 ± 8.80 years, with a predominance of male participants. More than three-fourths of the participants were overweight or obese, and hypertension was the most frequently reported comorbidity. Symptoms suggestive of TMD were identified in 52.7% of patients, with mild TMD being the most prevalent category. No statistically significant associations were observed between TMD severity and age, gender, or BMI. Among OSA-related symptoms, excessive daytime sleepiness demonstrated a statistically significant association with TMD severity (p < 0.05), whereas snoring, witnessed apneas, morning headache, and non-restorative sleep did not show significant associations.

Conclusion

A relatively high prevalence of symptoms suggestive of TMD was observed among patients with severe OSA. TMD severity showed no significant association with most demographic or anthropometric variables. Although excessive daytime sleepiness was associated with TMD severity, this finding should be interpreted cautiously, given the cross-sectional design. These results represent descriptive observations, and further comparative and longitudinal studies are required to clarify the relationship between OSA-related symptoms and TMD.

## Introduction

Obstructive sleep apnea (OSA) is a highly prevalent sleep-related breathing disorder characterized by recurrent episodes of partial or complete upper airway obstruction during sleep, leading to intermittent hypoxia, sleep fragmentation, and excessive daytime somnolence. OSA has been implicated in a wide range of systemic consequences, including cardiovascular disease, metabolic dysfunction, neurocognitive impairment, and reduced quality of life. Epidemiological evidence indicates that OSA affects a substantial proportion of the adult population worldwide, underscoring its growing public health significance [1]. The diagnosis of OSA is established by polysomnography, which remains the gold standard for quantifying disease severity using the apnea-hypopnea index (AHI) [2].

Temporomandibular disorder (TMD) represents a heterogeneous group of musculoskeletal conditions that involve the temporomandibular joint (TMJ), masticatory muscles, and associated craniofacial structures. Clinically, TMD is characterized by orofacial pain, joint sounds, restricted mandibular movement, and functional limitations, which may adversely affect daily activities and overall well-being. Studies evaluating patients with sleep-disordered breathing have reported a higher prevalence of TMD signs and symptoms among individuals with OSA, compared with those without the disorder [3].

A systematic review examining the relationship between sleep disorders and TMDs demonstrated a consistent association between impaired sleep quality and the presence of TMD, suggesting that sleep disturbance may play an important role in the development or exacerbation of temporomandibular dysfunction [4]. Further observational evidence has corroborated these findings, showing a significantly increased prevalence of TMD symptoms in patients with untreated OSA [5].

The pathophysiological mechanisms underlying the relationship between OSA and TMD are likely multifactorial. Sleep fragmentation and recurrent arousals in OSA have been associated with altered pain modulation, central sensitization, and increased musculoskeletal pain sensitivity. In addition, intermittent hypoxia promotes systemic inflammation through elevated levels of pro-inflammatory cytokines, which may contribute to joint and muscle pathology. Recent genetic evidence, derived from bidirectional Mendelian randomization analysis, has suggested a unidirectional causal relationship, indicating that OSA increases the risk of developing TMD, while TMD does not independently increase the risk of OSA [6].

Evidence from large-scale cohort studies, including the Orofacial Pain: Prospective Evaluation and Risk Assessment (OPPERA) study, has highlighted an overlap between symptoms of sleep-disordered breathing and those related to TMDs [7]. However, most available studies focus on associations or potential causal relationships, rather than quantifying the burden of TMD-related symptoms in well-characterized patients with OSA. Furthermore, limited multicentric data exist evaluating the prevalence of screened TMD symptoms in patients with polysomnography-confirmed OSA, particularly when standardized screening tools, such as the Fonseca Anamnestic Index (FAI), are used.

Therefore, the present cross-sectional, multicentric study was designed to assess the burden and prevalence of self-reported TMD symptoms in patients with polysomnography-confirmed severe OSA, using the FAI, rather than to investigate causal mechanisms between the two conditions. Additionally, the study evaluated the association between TMD severity and selected demographic and clinical variables, including age, gender, body mass index (BMI), and presenting sleep-related symptoms.

## Materials and methods

Study design

This cross-sectional, multicentric, observational study was conducted from August 2024 to August 2025 to assess the prevalence of TMDs among patients diagnosed with OSA. The study was carried out at the Department of Prosthodontics and Crown & Bridge, Sardar Patel Post Graduate Institute of Dental and Medical Sciences (SPPGIDMS), Lucknow, India, in collaboration with the Department of Prosthodontics and Crown & Bridge, King George's Medical University (KGMU), and the Department of Respiratory Medicine, KGMU, Lucknow, India.

Ethical considerations

The study protocol was reviewed and approved by the Research Advisory Council (Ref. No.: PROSTHO/06/232436/RAC; dated February 10, 2024) and the Institutional Ethics Committee (Ref. No.: PROSTHO/06/232436/IEC; dated February 10, 2024) of SPPGIDMS, Lucknow. The study was conducted in accordance with the ethical principles outlined in the Declaration of Helsinki and applicable institutional guidelines.

Written informed consent was obtained from all participants before enrollment. Participant confidentiality was strictly maintained, and all data were anonymized to ensure privacy. Participation was entirely voluntary, and participants were informed of their right to withdraw from the study at any stage, without any consequences. As the study was observational in nature, no invasive procedures were performed, thereby posing minimal risk to the participants.

Study population

The target population comprised adult patients diagnosed with OSA based on overnight polysomnography. Severe OSA (AHI ≥30 events/hour) is associated with a higher burden of systemic comorbidities and more pronounced physiological alterations; therefore, only patients with severe OSA were included in this study to evaluate TMD characteristics in individuals with greater disease severity and to maintain a homogeneous study population [8-10]. Both male and female participants, aged 18-60 years, were considered eligible for inclusion. 

Inclusion criteria

Participants aged between 18 and 60 years, who were newly diagnosed with severe OSA, were included in the study. The diagnosis of OSA was established exclusively through overnight polysomnography, and severe OSA was defined by an AHI of ≥30 events per hour [11]. Only patients with no prior history of facial trauma or maxillofacial surgical interventions were considered eligible for inclusion.

For descriptive purposes, OSA severity classification based on AHI values was as follows: mild (5-14 events/hour), moderate (15-29 events/hour), and severe (≥30 events/hour) [11].

Exclusion criteria

Patients were excluded from the study if they had been previously diagnosed with, or were undergoing treatment for, OSA at the time of recruitment. Individuals with a current or past history of treatment for TMD, TMJ fractures, or TMJ surgeries were also excluded to avoid the influence of prior therapeutic interventions on symptom reporting. Additional exclusion criteria included the use of analgesic medications within the preceding five days, ongoing antidepressant therapy, and a history of neurological or neuropathic disorders.

Participants presenting with malocclusion, or a history of orthodontic treatment (past or ongoing), were excluded because these conditions may independently influence TMJ function and TMD-related symptoms, potentially acting as confounding factors in the assessment of symptom burden. Immunocompromised individuals, syndromic patients, non-cooperative participants, and those who declined to provide written informed consent were also excluded from the study.

Sample size calculation

The sample size was calculated to estimate the prevalence of TMD symptoms among patients with severe OSA. Based on a previous cross-sectional study by Bartolucci et al., which reported a prevalence of TMD symptoms of 46.5% among adult OSA patients, a similar prevalence was assumed for the present study [12]. The sample size calculation was therefore performed to estimate the prevalence of TMD symptoms within the population of severe OSA patients, rather than to compare prevalence across different OSA severity categories.

The sample size was calculated using the formula suggested by Charan and Biswas [13]: $n = \frac{(Z_{1-\alpha/2})^{2} \times p \times (1 - p)}{d^{2}}$, where \( n = \text{required sample size} \), $Z_{1-\alpha/2} = 1.96 \quad \text{(for a 95\% confidence interval)}$, $p = \text{expected prevalence} = 0.47$, $d = \text{absolute precision} = 0.1$. Substituting the values into the formula: $n = \frac{(1.96)^2 \times 0.47 \times (1 - 0.47)}{(0.1)^2}$, $n = 92.19$. The minimum required sample size was rounded to 93 participants. Considering a 10% allowance for potential data loss, the final sample size was increased and rounded off to 110 participants.

Sampling method

**Figure 1:**
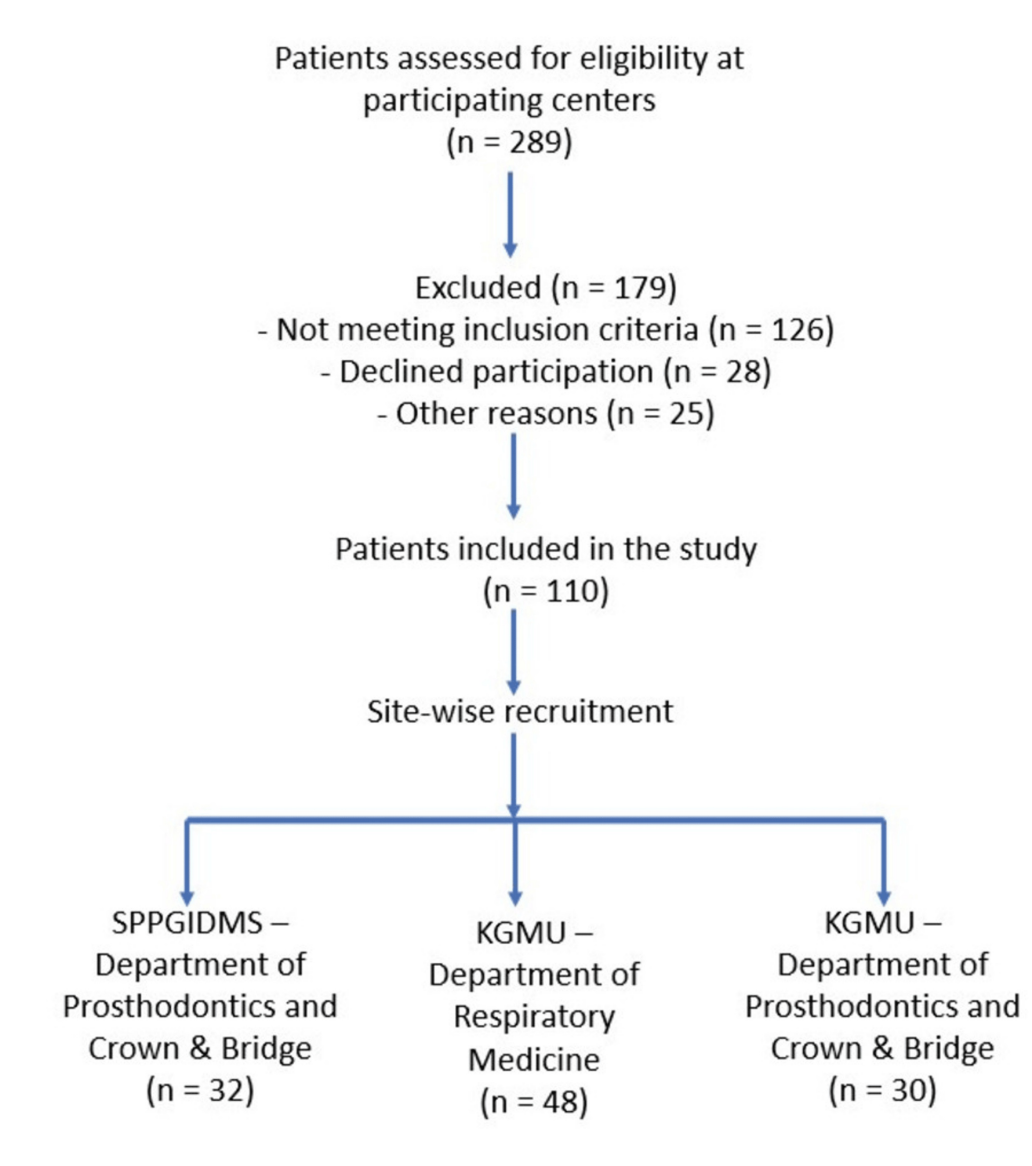
Participant recruitment flow diagram A flow diagram shows the recruitment and site-wise distribution of patients with obstructive sleep apnea included in the study across participating centers (total n = 110).

A total of 110 patients diagnosed with severe OSA were consecutively recruited from three participating centers: the Department of Prosthodontics and Crown & Bridge, SPPGIDMS; the Department of Respiratory Medicine, KGMU; and the Department of Prosthodontics and Crown & Bridge, KGMU. A recruitment flow diagram illustrating the screening process, exclusions, and site-wise distribution of enrolled participants is shown in Figure 1. No control group was included, as the objective of the study was to determine the prevalence of screened TMD symptoms within the OSA population.

To ensure consistency across the participating centers, data collection procedures were standardized and conducted by trained investigators following a uniform protocol. Patients with polysomnography-confirmed severe OSA were consecutively identified from outpatient clinics at each site. Anthropometric and clinical variables, including age, gender, and BMI, were obtained from clinical records and verified during patient evaluation. Participants with incomplete questionnaire responses or missing clinical data were excluded from the final analysis.

Study tool and data collection

All eligible participants were evaluated for symptoms suggestive of TMD using the FAI [11]. After obtaining written informed consent, participants were asked to complete the structured questionnaire. The FAI, originally described by Fonseca et al., consists of 10 self-reported questions with three response options: “yes” (10 points), “sometimes” (5 points), and “no” (0 points). The total score ranges from 0 to 100, and the severity classification is as follows: no TMD (0-15), mild TMD (20-40), moderate TMD (45-65), and severe TMD (70-100) [14].

For the present study, the questionnaire was administered primarily as a self-completed instrument. However, for participants with limited literacy, the questions were read aloud and clarified by the investigator to ensure accurate understanding while maintaining the original wording of the instrument. The questionnaire was provided in English and in a translated local-language version, which was reviewed by bilingual experts to ensure conceptual equivalence with the original instrument. Prior to the commencement of the study, the translated questionnaire was pilot tested on a small group of participants to assess clarity, comprehension, and feasibility of administration, and no modifications were required following pilot testing. No structural modifications were made to the original instrument.

Data collection procedure

Eligible participants were consecutively recruited from sleep clinics, prosthodontic departments, and general outpatient departments. After obtaining written informed consent, participants were briefed about the objectives and procedures of the study. Data were collected using a structured case proforma that included demographic characteristics (age, sex, BMI, medical history, and lifestyle factors).

Body weight was measured using a calibrated digital weighing scale with participants wearing light clothing and no footwear, and height was measured using a stadiometer with the participant standing upright. BMI was calculated using the formula [15]: \( BMI = \frac{\text{Weight (kg)}}{\text{Height (m)}^2} \).

The severity of OSA is classified as mild, moderate, or severe, based on polysomnography reports and assessment of TMDs using the FAI. All collected data were compiled and securely entered into a password-protected database for subsequent analysis.

Statistical analysis

Statistical analysis was performed using IBM SPSS Statistics for Windows, Version 21 (Released 2012; IBM Corp., Armonk, NY, USA). Descriptive statistics were presented as frequencies and percentages for categorical variables, and as mean ± standard deviation for continuous variables. The prevalence of symptoms suggestive of TMD was calculated with 95% confidence intervals (CIs). The normality of continuous variables was assessed using the Shapiro-Wilk test (and the Kolmogorov-Smirnov test where appropriate). Associations between categorical variables were evaluated using the Chi-square test, while comparisons of mean values among groups were performed using analysis of variance (ANOVA), where applicable. A two-tailed p-value < 0.05 was considered statistically significant.

As the objective of the study was to estimate the prevalence and distribution of screened TMD symptoms in patients with severe OSA, advanced modeling techniques, such as ordinal or multinomial regression, were not performed. Severity categories of TMD were, therefore, analyzed descriptively.

## Results

A total of 110 patients diagnosed with OSA through polysomnography were enrolled in the study. The following table shows the demographic profile of patients enrolled in the study. The age of the patients ranged from 25 to 60 years, with a mean age of 48.28 ± 8.80 years. Only 12 (10.9%) of patients were aged 25-35 years; the largest group was aged 46-55 years; 24 (21.8%) of patients were aged >55 years; and the remaining 25 (22.7%) were aged 36-45 years. A dominance of the male gender (74, 67.3%) was observed in the study, while only 36 (32.7%) of patients were female (Table 1).

**Table 1:** Demographic profile of study population (n = 110)

Age in years	Number (n)	Percentage (%)
25-35 years	12	10.9
36-45 years	25	22.7
46-55 years	49	44.5
>55 years	24	21.8
Mean age ± SD (in years)	48.28 ± 8.80	
Gender	Number (n)	Percentage (%)
Male	74	67.3
Female	36	32.7

The range of height, weight, and BMI of the study population was 150-184 cm, 55-99 kg, and 18.8-43.1 kg/m², respectively. The mean height, weight, and BMI of patients were 165.57 ± 7.33 cm, 81.29 ± 10.07 kg, and 29.80 ± 4.45 kg/m², respectively (Table 2).

**Table 2:** Anthropometric parameters of study population (n = 110)

Parameter	Number (n)	Minimum	Maximum	Mean	SD
Height (cm)	110	150	184	165.57	7.33
Weight (kg)	110	55	99	81.29	10.07
BMI (kg/m^2^)	110	18.8	43.1	29.8	4.45

Out of 110 patients enrolled in the study, 49 (44.5%) were obese (BMI ≥30 kg/m²), 47 (42.7%) were overweight (BMI 25-29.9 kg/m²), and 14 (12.7%) had a normal BMI (18.5-24.9 kg/m²) (Table 3).

**Table 3:** Distribution of study population according to body mass index

BMI	Number of cases (n)	Percentage
Normal (18.5-24.9 kg/m²)	14	12.7
Overweight (25-29.9 kg/m²)	47	42.7
Obese (≥30 kg/m²)	49	44.5

Out of 110 patients enrolled in the study, 28 (25.5%) had no comorbidity. The most common comorbidity was hypertension (46, 41.8%), followed by diabetes mellitus (31, 28.2%), dyslipidemia (28, 25.5%), and hypothyroidism (8, 7.3%) (Table 4).

**Table 4:** Comorbidities in study population

Comorbidities	Number of cases (n)	Percentage
Diabetes mellitus	31	28.2
Dyslipidemia	28	25.5
Hypertension	46	41.8
Hypothyroid	8	7.3
No comorbidity	28	25.5

Only 28 (25.5%) of patients had no comorbidity, 55 (50.0%) had a single comorbidity, and the remaining 27 (24.5%) had multiple comorbidities (Table 5).

**Table 5:** Comorbidity status in study population

Status	Number of cases (n)	Percentage
No comorbidity	28	25.5
Single comorbidity	55	50.0
Multiple comorbidities	27	24.5

Patients presented with multiple symptoms. The most common presenting symptom was daytime sleepiness (73, 66.4%), followed by witnessed apneas (68, 61.8%) and non-restorative sleep (66, 60.0%). The least common symptom was morning headache (56, 50.9%), followed by snoring (60, 54.5%) (Table 6).

**Table 6:** Presenting symptoms in study population

Symptom	Number of cases (n)	Percentage
Daytime sleepiness	73	66.4
Morning headache	56	50.9
Snoring	60	54.5
Witnessed apneas	68	61.8
Non-restorative sleep	66	60.0

Out of 110 patients with sleep apnea enrolled in the study, the prevalence of TMD was 58 (52.7%) patients: 11 (10.0%) had severe TMD, 19 (17.3%) had moderate TMD, and 28 (25.5%) had mild TMD (Table 7).

**Table 7:** Prevalence and severity of temporomandibular disorder in study population

Temporomandibular disorder	Number of cases (n)	Percentage
Temporomandibular disorder absent	52	47.3
Temporomandibular disorder present	58	52.7
Mild temporomandibular disorder	28	25.5
Moderate temporomandibular disorder	19	17.3
Severe temporomandibular disorder	11	10

Patients with severe TMD were younger (46.73 ± 9.07 years) compared to those with no TMD, mild TMD, and moderate TMD (47.85 ± 9.14, 48.68 ± 8.97, and 49.79 ± 7.78 years), but this difference was not statistically significant (Table 8).

**Table 8:** Association of severity of temporomandibular disorder with age F = 0.356; p = 0.785 (not significant)

Temporomandibular disorder severity	Number of cases	Min	Max	Mean	SD
No temporomandibular disorder	52	25	60	47.85	9.14
Mild temporomandibular disorder	28	26	60	48.68	8.97
Moderate temporomandibular disorder	19	34	60	49.79	7.78
Severe temporomandibular disorder	11	34	59	46.73	9.07

Dominance of the male gender was observed overall in 74 (67.3%) patients, as well as in all groups: no TMD (34, 65.4%), mild TMD (20, 71.4%), moderate TMD (13, 68.4%), and severe TMD (7, 63.6%). The proportion of females was higher in severe TMD (4, 36.4%) and no TMD (18, 34.6%) compared to patients with mild TMD (8, 28.6%) and moderate TMD (6, 31.6%), but this difference was not statistically significant (Table 9).

**Table 9:** Association of severity of temporomandibular disorder with gender χ² = 0.381; p = 0.944 (not significant)

Gender	Total (N = 110)	No temporomandibular disorder (n = 52)		Mild temporomandibular disorder (n = 28)		Moderate temporomandibular disorder (n = 19)		Severe temporomandibular disorder (n = 11)	
		No.	%	No.	%	No.	%	No.	%
Male	74	34	65.4	20	71.4	13	68.4	7	63.6
Female	36	18	34.6	8	28.6	6	31.6	4	36.4

The proportion of patients with normal BMI was higher among those with no TMD (9, 17.3%), as compared to patients with mild TMD (2, 7.1%), moderate TMD (2, 10.5%), and severe TMD (1, 9.1%). Patients categorized as overweight were higher among severe TMD (7, 63.6%), as compared to no TMD (22, 42.3%), mild TMD (11, 39.3%), and moderate TMD (7, 36.8%); the rest of the patients were obese. The association of BMI category with the severity of TMD was not found to be statistically significant (Table 10).

**Table 10:** Association of severity of temporomandibular disorder with BMI category χ² = 4.819; p = 0.567 (not significant)

BMI Category	Total (N = 110)	No temporomandibular disorder (n = 52)		Mild temporomandibular disorder (n = 28)		Moderate temporomandibular disorder (n = 19)		Severe temporomandibular disorder (n = 11)	
		No.	%	No.	%	No.	%	No.	%
Normal	14	9	17.3	2	7.1	2	10.5	1	9.1
Overweight	47	22	42.3	11	39.3	7	36.8	7	63.6
Obese	49	21	40.4	15	53.6	10	52.6	3	27.3

The severity of TMD showed a statistically significant association with daytime sleep symptoms only. Daytime sleep was reported by a significantly lower proportion of patients with severe TMD (4, 36.4%), as compared to patients with no TMD (39, 75.0%), moderate TMD (15, 78.9%), and mild TMD (15, 53.6%).

Morning-time headache had been reported by a higher proportion of patients with mild TMD (18, 64.3%), and moderate TMD (10, 52.6%), as compared to those with no TMD (23, 44.2%), and severe TMD (5, 45.5%), but this difference was not found to be statistically significant.

A higher proportion of patients with severe TMD (7, 63.6%), and moderate TMD (12, 63.2%), as compared to no TMD (25, 48.1%), and mild TMD (16, 57.1%), presented with the symptom of snoring; this difference was not found to be statistically significant.

A higher proportion of patients with moderate TMD (13, 68.4%) and severe TMD (7, 63.6%), as compared to no TMD (32, 61.5%), and mild TMD (16, 57.1%), witnessed apneas; this difference was not found to be statistically significant.

Non-rest sleep had been reported in a higher proportion of patients with severe TMD (9, 81.8%), as compared to patients with no TMD (32, 61.5%), mild TMD (15, 53.6%), and moderate TMD (10, 52.6%); this difference was not found to be statistically significant.

The prevalence of TMD among patients with OSA was 52.7%; TMD did not show any significant association with age, gender, BMI category, or most of the presenting symptoms. Daytime sleepiness was observed in a significantly lower proportion of severe TMD patients as compared to no TMD and mild-to-moderate TMD patients.

The distribution of OSA-related symptoms across TMD severity categories did not demonstrate a consistent linear gradient. The frequencies varied across groups, likely reflecting differences in subgroup sizes. Therefore, the results presented should be interpreted as descriptive comparisons rather than evidence of a severity-dependent trend (Table 11).

**Table 11:** Association of severity of temporomandibular disorder with presenting symptoms Distribution of obstructive sleep apnea-related symptoms across temporomandibular disorder severity categories among patients with obstructive sleep apnea. Values are presented as frequency and percentage within each severity group. Statistical comparisons were performed using the Chi-square test, and a p-value < 0.05 was considered statistically significant.

Presenting symptom	Total (N = 110)	No temporomandibular disorder (n = 52)		Mild temporomandibular disorder (n = 28)		Moderate temporomandibular disorder (n = 19)		Severe temporomandibular disorder (n = 11)		Statistical significance	
		No.	%	No.	%	No.	%	No.	%	χ²	p
Daytime sleep	73	39	75.0	15	53.6	15	78.9	4	36.4	9.573	0.023
Morning headache	56	23	44.2	18	64.3	10	52.6	5	45.5	3.086	0.379
Snoring	60	25	48.1	16	57.1	12	63.2	7	63.6	1.889	0.596
Witnessed apneas	68	32	61.5	16	57.1	13	68.4	7	63.6	0.627	0.890
Non-rest sleep	66	32	61.5	15	53.6	10	52.6	9	81.8	3.145	0.370

## Discussion

The present multicentric, cross-sectional study evaluated the prevalence and clinical correlates of TMDs among polysomnography-confirmed OSA patients in an Indian population. Using the FAI, TMD was identified in 52.7% of the study cohort, indicating a substantial coexistence of temporomandibular dysfunction in individuals with sleep-disordered breathing. The observed prevalence of symptoms suggestive of TMD in patients with severe OSA highlights a notable burden of screened TMD symptoms in this population. However, as the present study employed a cross-sectional design without a control group, the findings should be interpreted as descriptive estimates of symptom prevalence rather than evidence of comparative or causal relationships.

Wu et al. reported an increased occurrence of TMD among individuals with OSA in a large, population-based cohort study [16]. These findings provide epidemiological context for the relatively high prevalence of screened TMD symptoms observed in the present study.

Influence of demographic and anthropometric factors

In the present study, age did not show a significant association with TMD severity. This finding contrasts with idiopathic TMD literature but aligns with observations from OSA-focused cohorts, where age did not demonstrate a significant association with TMD severity in this cohort. Similarly, gender did not significantly influence TMD severity. Although idiopathic TMD exhibits female predominance, the male predominance of OSA may attenuate sex-related differences in temporomandibular pathology. These observations are consistent with population-based findings reported by Wu et al. [16].

BMI, despite being a major risk factor for OSA, did not significantly affect TMD severity. BMI did not show a significant association with TMD severity in this cohort, suggesting that the relationship between obesity and temporomandibular symptoms may be complex and requires further investigation, further emphasizing the role of sleep-related functional disturbances in OSA-associated TMD.

Therapeutic considerations related to oral appliance therapy

The relationship between OSA treatment and temporomandibular health is clinically relevant. Doff et al. evaluated long-term oral appliance therapy and reported that mandibular advancement devices may induce mild and transient temporomandibular side effects; however, clinically significant TMJ deterioration was uncommon [17]. These findings indicate that adaptive neuromuscular remodeling occurs over time in most patients undergoing oral appliance therapy.

Role of bruxism and parafunctional activity

Sleep bruxism represents an important behavioral link between OSA and TMD. Massahud et al. demonstrated a significant association between sleep bruxism and OSA, highlighting the contribution of nocturnal arousals and altered central neurotransmission to parafunctional activity [18]. An evidence-based review by Balasubramaniam et al. further emphasized that sleep-disordered breathing can trigger repetitive masticatory muscle activation during micro-arousals, increasing the risk of muscle fatigue and TMJ overload [19].

Sleep disturbance, pain modulation, and TMD

Sleep fragmentation and deprivation play a critical role in pain perception. Experimental evidence has shown that sleep deprivation significantly impairs endogenous pain inhibition and increases spontaneous pain sensitivity, providing a neurophysiological explanation for enhanced orofacial pain in sleep-disordered individuals [20]. These mechanisms likely contribute to increased TMD severity among OSA patients experiencing chronic sleep disruption.

Imaging evidence linking OSA and mandibular biomechanics

Dynamic imaging studies have provided further insight into the relationship between OSA and mandibular function. Moon et al. demonstrated that sleep magnetic resonance imaging can identify functional airway collapse and mandibular positional changes during sleep, underscoring the biomechanical interdependence between airway patency and mandibular posture [21]. Such alterations may have long-term implications for TMJ loading and dysfunction.

Daytime sleepiness and oral behaviors

Clinical evidence suggests that excessive daytime sleepiness is closely associated with adverse oral behaviors. Xiang et al. reported that increased daytime sleepiness correlates with heightened parafunctional oral activity among patients with TMD, supporting the role of sleep-related fatigue in exacerbating masticatory muscle overuse [22]. This finding aligns with the present study, where daytime somnolence emerged as a significant correlate of TMD severity.

Sleep quality in chronic TMD

Further reinforcing this association, Lee et al. demonstrated that patients with chronic TMD exhibit significantly poorer sleep quality compared with healthy controls, highlighting the bidirectional relationship between sleep disturbance and temporomandibular pain [23]. These findings suggest that sleep quality assessment should be an integral component of TMD evaluation.

Long-term dental effects of oral appliance therapy

In addition to temporomandibular effects, long-term oral appliance therapy has been associated with dental changes. Doff et al. reported that although dental side effects, such as occlusal changes, may occur, they are generally mild and clinically manageable with regular follow-up [24]. These findings support the continued use of oral appliances in appropriately selected OSA patients.

Comparative effects of OSA treatment modalities

More recently, Attia et al., in a randomized clinical trial, compared the effects of continuous positive airway pressure (CPAP) and oral appliance therapy on TMJ health and found no significant deterioration in TMJ status with either treatment modality [25]. In some cases, improvement in TMJ symptoms was observed, likely due to reduced nocturnal hypoxia and muscle hyperactivity.

Clinical implications of the study

The high prevalence of TMD symptoms among patients with severe OSA suggests the need for routine screening of TMD in individuals diagnosed with OSA. Early identification of TMD symptoms may facilitate timely referral to dental specialists and allow for multidisciplinary management involving sleep physicians and dental professionals. Furthermore, recognizing the potential coexistence of OSA and TMD may help improve patient outcomes, treatment planning, and overall quality of life by addressing both conditions simultaneously.

Limitations of the study

This study has several limitations that should be considered when interpreting the findings. First, the cross-sectional design precludes the establishment of causal relationships between OSA and TMDs; therefore, the results should be interpreted as descriptive associations rather than evidence of temporality or causation. Second, the study population consisted exclusively of patients with severe OSA, which prevented comparisons across different OSA severity categories. Third, the absence of a control group without OSA limits the ability to determine whether the observed prevalence of screened TMD symptoms differs from that of comparable populations without sleep-disordered breathing.

Although the study was conducted across multiple tertiary care centers, participants were recruited from hospital-based settings, which may limit the generalizability of the findings to the broader community population. In addition, TMD assessment was performed using the FAI, a screening instrument rather than a comprehensive clinical or imaging-based diagnostic protocol, which may introduce reporting bias. Potential confounding variables, including psychological stress, parafunctional habits, and inflammatory markers, were not evaluated. Finally, while the sample size was adequate for estimating prevalence, it may have limited the statistical power to detect subtle associations between clinical variables.

Future multicentric, longitudinal studies incorporating control groups and objective diagnostic assessments are required to better elucidate the relationship between OSA-related symptoms and temporomandibular dysfunction.

## Conclusions

This cross-sectional study identified a relatively high prevalence of symptoms suggestive of TMD among patients with severe OSA. TMD severity was not significantly associated with age, gender, BMI, or most OSA-related symptoms. Excessive daytime sleepiness showed a statistically significant association with TMD severity; however, this observation should be interpreted cautiously, given the cross-sectional design and the observed distribution across severity groups. Overall, these findings represent descriptive observations of screened TMD symptoms in patients with severe OSA. Further well-designed, comparative, and longitudinal studies are required to better clarify the relationship between OSA-related symptoms and temporomandibular dysfunction.
